# Pyrotinib Combined With Vinorelbine in HER2-Positive Metastatic Breast Cancer: A Multicenter Retrospective Study

**DOI:** 10.3389/fonc.2021.664429

**Published:** 2021-04-20

**Authors:** Yi Li, Yixuan Qiu, Huihui Li, Ting Luo, Wei Li, Hong Wang, Bin Shao, Biyun Wang, Rui Ge

**Affiliations:** ^1^ Huadong Hospital Affiliated to Fudan University, Shanghai, China; ^2^ Department of Medical Oncology, Fudan University Shanghai Cancer Center, Department of Oncology, Shanghai Medical College, Fudan University, Shanghai, China; ^3^ Department of Breast Medical Oncology, Shandong Cancer Hospital and Institute, Shandong First Medical University and Shandong Academy of Medical Sciences, Jinan, China; ^4^ Department of Head, Neck and Mammary Gland Oncology, Cancer Center, West China Hospital, Sichuan University, Chengdu, China; ^5^ Department of Medical Oncology, Jiangsu Province Hospital, Nanjing, China; ^6^ Department of Breast Oncology, The Third Hospital of Nanchang, Nanchang, China; ^7^ Department of Breast Oncology, Key Laboratory of Carcinogenesis and Translational Research (Ministry of Education), Peking University Cancer Hospital & Institute, Beijing, China

**Keywords:** human epidermal growth factor receptor 2 positive, metastatic breast cancer, pyrotinib, vinorelbine, tyrosine kinase inhibitor

## Abstract

**Introduction:**

Pyrotinib plus capecitabine has been approved in China for human epidermal growth factor receptor 2 (HER2)-positive metastatic breast cancer (MBC). Meanwhile, vinorelbine is another important chemotherapy option for MBC available in oral and intravenous forms. Thus, pyrotinib plus vinorelbine may represent a new treatment option, particularly for patients with failed capecitabine treatment. This study reported the first real-world data for pyrotinib plus vinorelbine therapy in HER2+ MBC.

**Methods:**

HER2+ MBC patients (n = 97) treated with pyrotinib plus vinorelbine in six institutions across China from May 2018 to June 2020 were enrolled. Progression-free survival (PFS), objective response rate (ORR), overall survival (OS), and toxicity profiles were determined.

**Results:**

Sixty-seven percent of patients received more than two lines of systematic therapy. Nearly all patients (97.9%) had received trastuzumab and 50.5% were administered lapatinib. When combined with pyrotinib, 74.2% received oral and 25.8% received intravenous vinorelbine. Median PFS (mPFS) was 7.8 (range, 4.7–10.8) months for all patients. The mPFS in patients administered pyrotinib as second-line therapy and third-or-higher-line therapy were 12.0 and 6.4 months, respectively. Patients who received pyrotinib plus oral or intravenous vinorelbine had similar mPFS (7.8 vs. 6.4 months, p = 0.871). The 23 patients with brain metastases had mPFS of 6.3 (range, 3.4–9.2) months. Lapatinib-naïve patients had significantly longer PFS than lapatinib-treated patients (10.8 months vs. 5.6 months, p = 0.020). Median OS was not achieved. The ORR for 96 patients was 34.3%. Common grade 3 and 4 adverse events were diarrhea (22.7%), neutropenia (7.2%), and leukopenia (4.1%).

**Conclusions:**

Pyrotinib plus vinorelbine therapy demonstrated promising effects in HER2+ MBC with tolerable toxicity, particularly in patients with second-line treatment and without prior lapatinib treatment, as well as in patients with brain metastases. Oral vinorelbine is a useful alternative to the intravenous form when combined with pyrotinib.

**Clinical Trial Registration:**

[ClinicalTrials.gov], identifier [NCT04517305].

## Introduction

Approximately 15–20% of patients with breast cancer overexpress the human epidermal growth factor receptor 2 (HER2) oncogene (1). This type of breast cancer exhibits an aggressive clinical behavior with higher rates of recurrence and metastasis (1). With the development of trastuzumab, as well as other anti-HER2 agents, such as pertuzumab, lapatinib, ado-trastuzumab emtansine, neratinib, and trastuzumab deruxtecan (2–7), the treatment and outcome of patients with HER2 positive (HER2+) metastatic breast cancer (MBC) have significantly improved. However, country-specific peculiarities should be considered. Specifically, neratinib, ado-trastuzumab emtansine, and trastuzumab deruxtecan are not available in all regions of the world. In addition, resistance to anti-HER2 treatment remains a challenge (1). Therefore, the continued development of novel anti-HER2 agents to further improve the efficacy of the treatment is important.

Pyrotinib is a novel oral, irreversible pan-ErbB tyrosine kinase inhibitor (TKI) that potently targets HER1, HER2, and HER4 (8). In fact, administration of pyrotinib plus capecitabine exhibited clinically meaningful results and acceptable tolerability in patients with HER2+ MBC in phase I, phase II, and phase III studies (9–12). In an open-label, multicenter, randomized phase II study, pyrotinib plus capecitabine treatment significantly improved the objective response rate (ORR; 78.5% vs. 57.1%, *p* = 0.01) and prolonged median progression-free survival (mPFS; 18.1 months vs. 7.0 months, *p* < 0.001) compared to lapatinib plus capecitabine. Moreover, the PHENIX study, a double-blinded, multicenter, randomized phase III study, reported that pyrotinib plus capecitabine significantly prolonged mPFS (11.1 months vs. 4.1 months, *p* < 0.001) and increased ORR (68.6% vs. 16.0%, *p* < 0.001) compared to capecitabine monotherapy (11). Recently, the phase III PHOEBE study reported that pyrotinib plus capecitabine, significantly prolonged the mPFS by 5.7 months compared with lapatinib plus capecitabine (12.5 vs. 6.8 months, *p* < 0.0001), thereby verifying the phase II findings (12). Though the overall survival (OS) data were not mature, a strong trend was observed toward prolonged survival following administration of pyrotinib plus capecitabine (12). However, these previous studies only included patients with HER2+ MBC who had previously received treatment with no more than two lines of systematic therapy. Pyrotinib was approved for use in China in August 2018 as a second-line standard-of-care for HER2+ MBC due to the remarkable results reported in the abovementioned phase II study, and is currently in phase I clinical trial in the United States (13). However, capecitabine is a chemotherapy regimen frequently used used in routine clinical practice, and it may cause many patients to have failed capecitabine treatment before they are able to receive pyrotinib-based therapy. Moreover, the treatment effect of pyrotinib combined with other chemotherapy drugs remains unclear, thereby limiting a clinician’s selection of chemotherapy drugs.

Vinorelbine is a semi-synthetic, antimitotic, microtubule destabilizing drug that has been shown to be effective and well-tolerated for the treatment of MBC (14). Two forms of this compound, intravenous and oral, are available for clinical use. The oral presentation not only has acceptable comparable efficacy and safety to the intravenous form, but it also allows patients to maintain their quality of life, as evidenced by the well-established patient preference for an oral formulation (15, 16). Moreover, vinorelbine has demonstrated efficacy and tolerability in combination with trastuzumab, lapatinib, or neratinib in clinical studies for patients with HER2+ MBC (17–19), which provides rationale for the evaluation of vinorelbine in combination with other anti-HER2 agents, such as pyrotinib. Furthermore, it is necessary to investigate new therapeutic options for the pyrotinib-based treatment regimen, particularly for patients who have failed capecitabine treatment.

We, therefore, conducted this multicenter study, which is the first, to our knowledge, to evaluate the efficacy and safety of pyrotinib plus vinorelbine in real-world HER2+ MBC and to provide a theoretical basis for clinical practice.

## Methods

### Subjects and Study Design

This is a retrospective, multicenter study that enrolled patients with HER2+ MBC treated with pyrotinib plus vinorelbine at six medical institutions, including Fudan University Shanghai Cancer Center, Shandong Cancer Hospital and Institute, West China Hospital Sichuan University, Jiangsu Province Hospital, the Third Hospital of Nanchang City, and Peking University Cancer Hospital and Institute, from May 2018 to June 2020. The Ethics Committee and Institutional Review Board of Fudan University Shanghai Cancer Center approved this study. All investigations were conducted in accordance with the Declaration of Helsinki. Our research is registered at clinicaltrials.gov (04517305).

### Patients

The inclusion criteria for participants were as follows: female sex; age ≥ 18 years; histologically or cytologically confirmed MBC with documentation of HER2 overexpression (tumor tissue protein expression demonstrated by immunohistochemistry category 3+ or positive results of fluorescence *in situ* hybridization); at least one cycle of pyrotinib plus vinorelbine treatment starting from May 2018 to June 2020 in the six hospitals mentioned above; dequate hematological, hepatic, and renal functions; and complete medical records. No limits on the number of prior cytotoxic regimens for metastatic disease were set. All data were retrospectively collected from medical records and laboratory results of individual institutions and administered by Fudan University Shanghai Cancer Center.

### Treatment and Dose Modification

Patients were prescribed pyrotinib plus vinorelbine in routine clinical practice. The standard dosage of pyrotinib is 400 mg single dose orally per day. Patients were treated with 25 mg/m^2^ vinorelbine intravenously or 60 mg/m^2^ orally on days 1 and 8 of a 21-day cycle. Starting dose, dose modification, dose interruption, treatment discontinuation, combination therapy with anti-HER2 agents, and/or radiotherapy were determined by physicians’ choice based on previous clinical trial results, general health status, and willingness of patients.

### Outcomes

The primary end point was PFS, defined as the time from drug administration to tumor progression or death by any cause, regardless of whichever occurred first. Secondary endpoints included ORR, OS, and safety. ORR was defined as the proportion of patients with complete response (CR) or partial response (PR). OS was defined as the time period from initial treatment of pyrotinib plus vinorelbine to death by any cause, or last follow-up. Adverse events (AEs) were retrospectively collected based on a patient self-reporting system and by reviewing biochemical test results.

Tumor response assessments were accessed based on Response Evaluation Criteria in Solid Tumors (RECIST) 1.1 criteria by computed tomography (CT), magnetic resonance imaging, and physical examination. All AEs were graded by the National Cancer Institute Common Terminology Criteria for Adverse Events (CTCAE, 4.03).

### Statistical Analysis

The median (range) or percentage of patients was used to represent clinicopathologic characteristics. The Kaplan–Meier method was used to estimate PFS and OS. Additionally, the Cox proportional hazards model was used to estimate hazard ratios and corresponding 95% confidence intervals (CIs). A log-rank test was conducted to perform exploratory analyses using the following variables: age, hormone receptor status, disease-free interval (DFI), number of metastatic sites, visceral metastases, number of metastatic systematic therapy lines of pyrotinib plus vinorelbine, trastuzumab resistance status, and prior lapatinib treatment.

Trastuzumab resistance was defined according to that described by Wong et al., “as progression at first radiological reassessment at 8–12 weeks or within 3 months after first-line trastuzumab with or without chemotherapy in the metastatic setting or new recurrences diagnosed during or within 12 months after adjuvant trastuzumab.” (20) Meanwhile, trastuzumab refractoriness was defined “as disease progression after two or more lines of trastuzumab-containing regimens that initially achieved disease response or stabilization at first radiological assessment” (20).

Cox multivariate models were performed based on the univariate analyses results. Two-tailed CIs and P-values were obtained. *p* < 0.05 was considered to represent statistically significant differences. SPSS24.0 was used to perform all statistical analyses.

## Results

### Baseline Characteristics

A total of 97 HER2+ MBC patients treated with pyrotinib plus vinorelbine between May 2018 and June 2020 at six institutions were included in the study. Baseline characteristics are presented in **Table 1**. The median age of patients at diagnosis was 53 (range 26–74) years. Sixteen patients had *de novo* stage IV breast cancer (16.5%). Moreover, 41.2% of patients had more than three metastatic sites, with the three most common metastatic sites determined to be the lung (45.4%), bone (40.2%), and liver (35.1%). An additional 67.0% patients had visceral metastases, while 23 (23.7%) had brain metastases. Almost all patients had been exposed to anti-HER2 therapy, with 97.9% prescribed trastuzumab and 50.5% exposed to lapatinib. Furthermore, 67% of patients received two or more lines of systematic therapy before pyrotinib plus vinorelbine, representing a heavily pretreated group. These results suggest that in a real-world setting, patients receiving pyrotinib plus vinorelbine are more likely to be heavily pretreated.

**Table 1:** Patient characteristics at baseline.

Characteristics	Number of patients (%)(N = 97)
Median age (years, range)	53 (26–74)
Hormone receptor status	
Positive	43 (44.3)
Negative	54 (55.7)
Disease-free interval	
Primary metastatic	16 (16.5)
≤1 year	31 (32.0)
>1 year	50 (51.5)
Metastatic sites	
Lung	44 (45.4)
Liver	34 (35.1)
Bone	39 (40.2)
Brain	23 (23.7)
Number of metastatic sites	
1	29 (29.9)
2	28 (28.9)
≥3	40 (41.2)
Visceral metastases	
Yes	65 (67.0)
No	32 (33.0)
Lines of advanced systematic therapy of pyrotinib plus vinorelbine	
1	2 (2.0)
2	30 (31.0)
≥3	65 (67.0)
Trastuzumab Resistance Status	
Resistance	33 (34.0)
Refractoriness	59 (60.8)
Unknown	5 (5.2)
Prior HER2-targeted therapy	
Trastuzumab	95 (97.9)
Lapatinib	49 (50.5)
T-DM1	3 (3.1)
Pertuzumab	3 (3.1)

### Treatment Administration

A total of 92.8% patients started pyrotinib treatments at the standard 400 mg/day dose, while 7.2% patients initiated pyrotinib treatment at a 320 mg/d dose (**Table 2**). Additionally, 16 (16.5%) and 12 (12.4%) patients experienced dose reduction and treatment interruption of pyrotinib, respectively. Meanwhile, 74.2% patients were treated with oral vinorelbine and 25.8% received intravenous vinorelbine. Thirteen (13.4%) patients experienced dose reduction of vinorelbine due to AEs, while 6 (6.2%) patients interrupted vinorelbine treatment. No patients discontinued treatment permanently due to AEs.

**Table 2:** Treatment administration.

Pyrotinib plus vinorelbine treatment	Number of patients (%)(N = 97)
Pyrotinib	
Starting dosage (mg/day)	
400	90 (92.8)
320	7 (7.2)
Dose reduction (mg/day)	
400→320	15 (15.5)
400→320→240	1 (1.0)
Interruption of pyrotinib treatment due to AEs	12 (12.4)
Vinorelbine	
Dosage form	
Oral	72 (74.2)
Intravenous	25 (25.8)
Dose reduction	
Yes	13 (13.4)
No	84 (86.6)
Interruption of vinorelbine treatment due to AEs	6 (6.2)

AEs, adverse events.

### Efficacy

**Figure 1:**
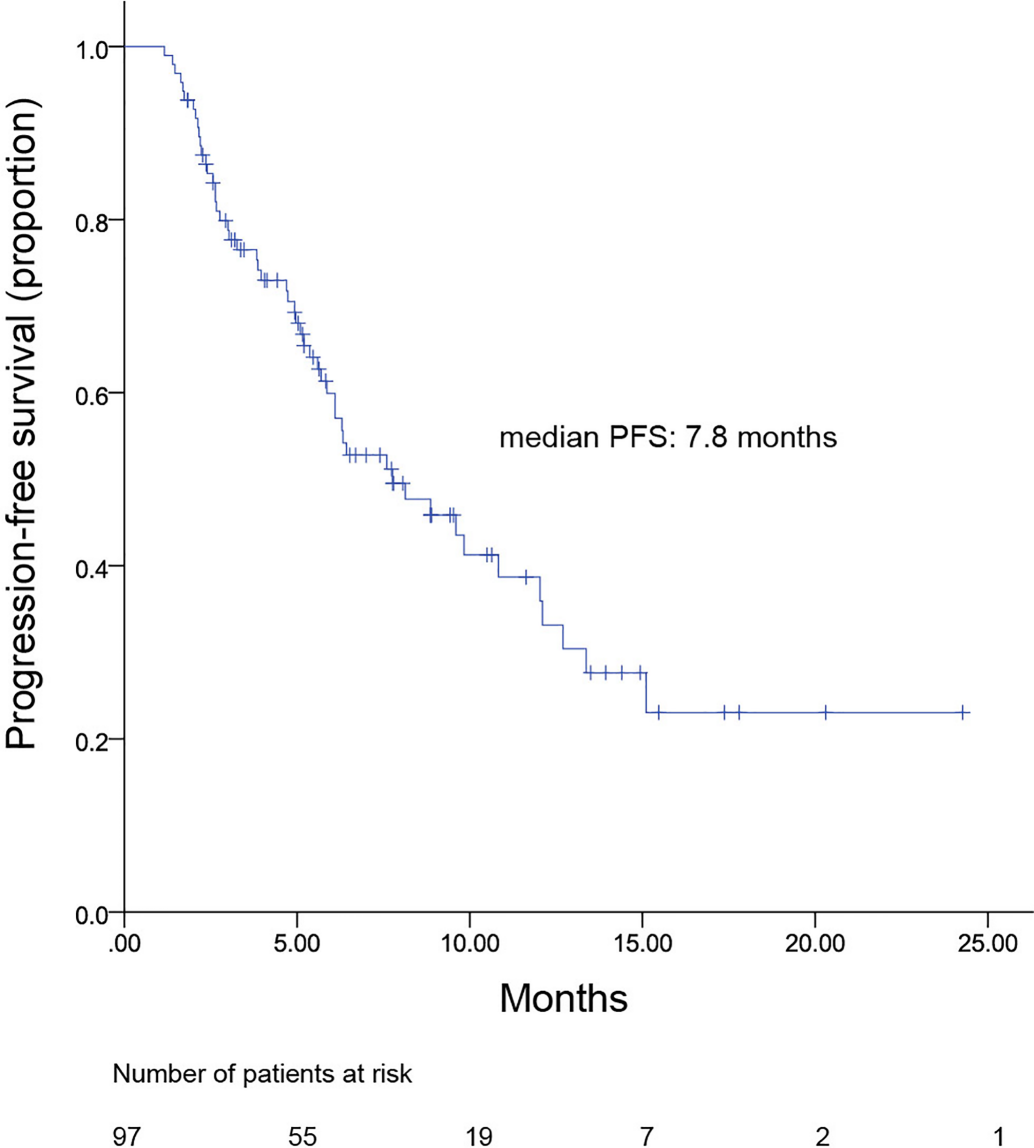
Kaplan–Meier curve for progression-free survival of all patients.

**Figure 2:**
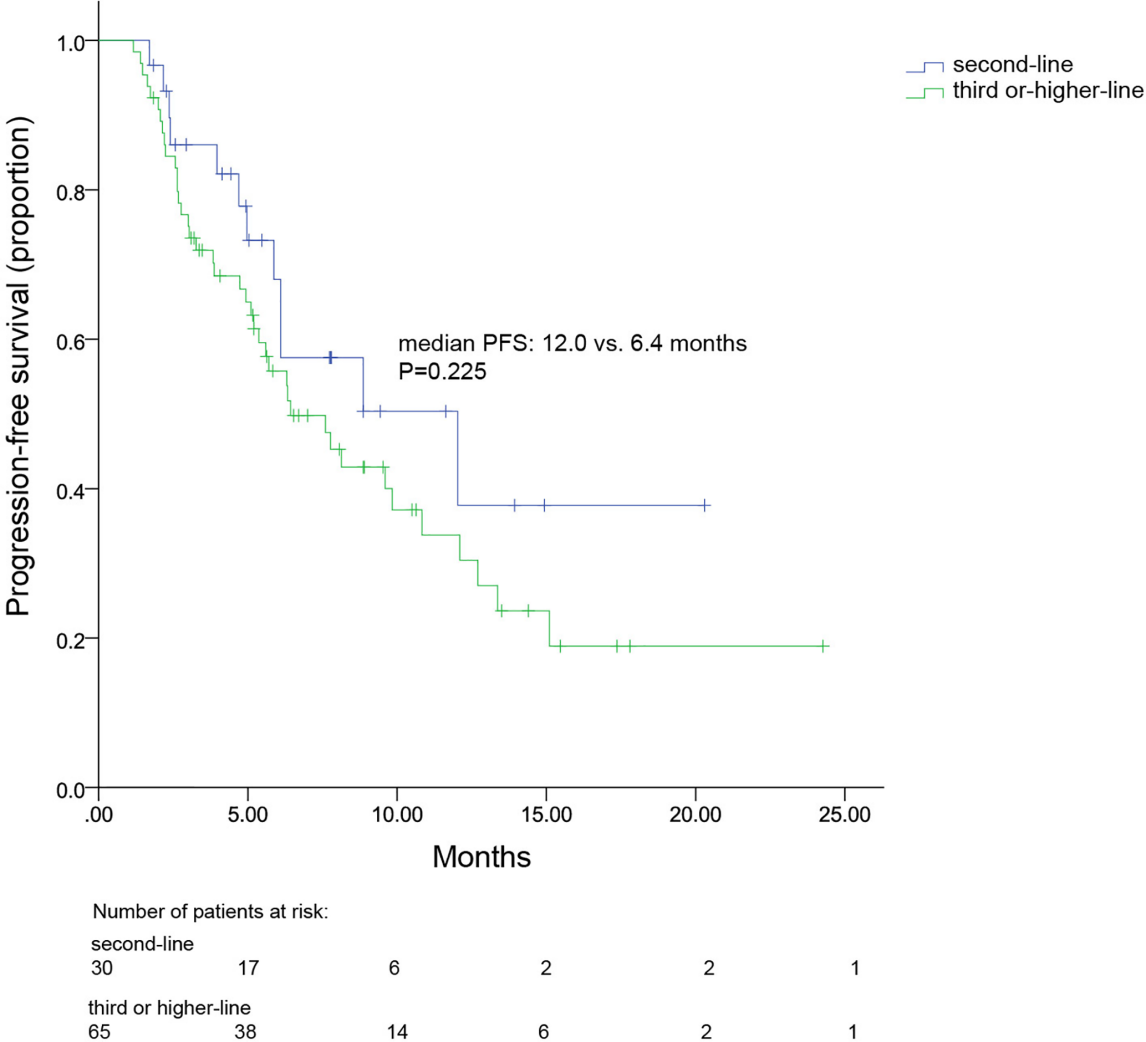
Kaplan–Meier curve for progression-free survival of patients receiving pyrotinib plus vinorelbine as their second-line or third-or-higher-line treatment.

**Figure 3:**
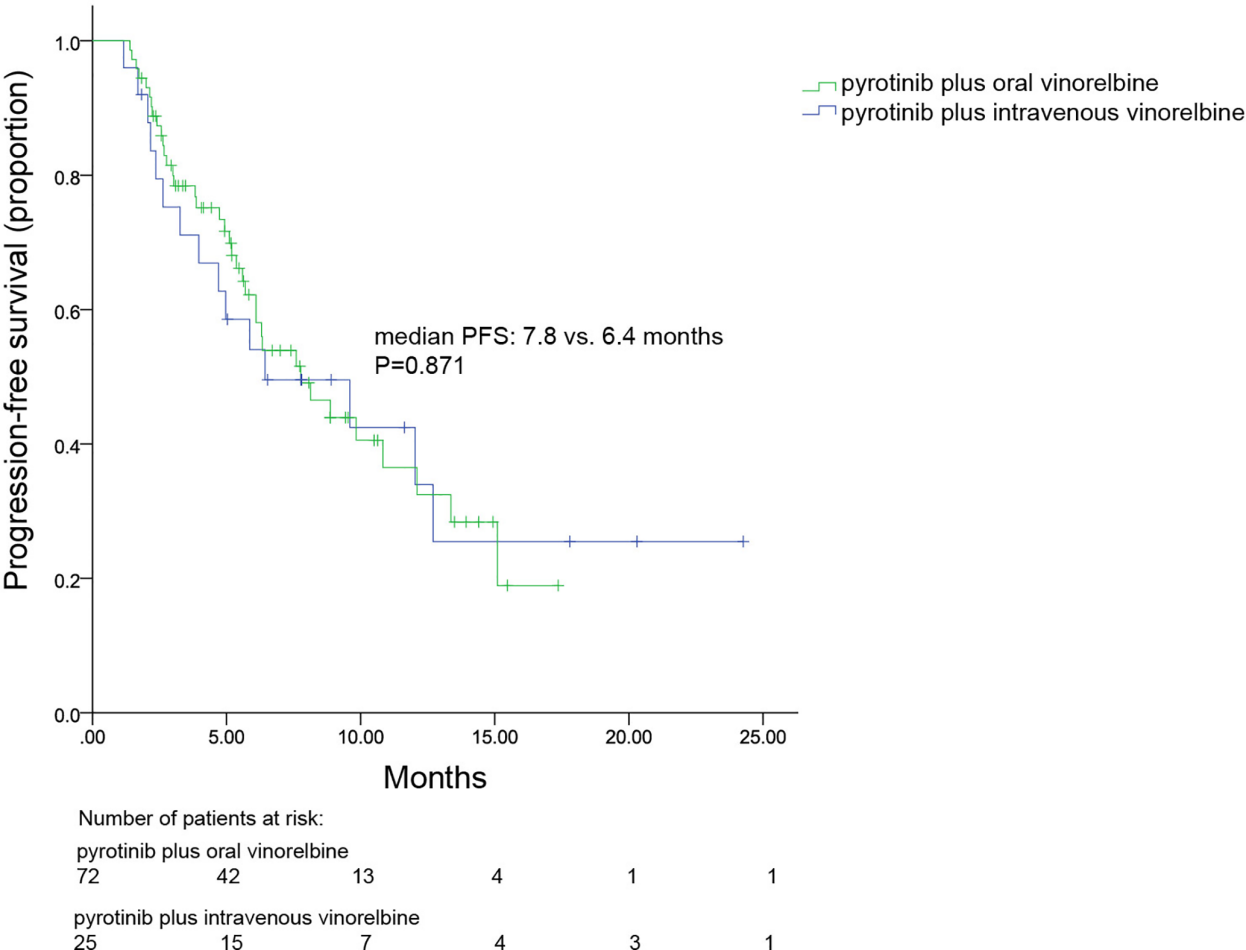
Kaplan–Meier curve for progression-free survival of patients receiving pyrotinib plus oral or intravenous vinorelbine.

All patients were included in PFS analysis. At a median follow-up of 8.7 months, 52 patients experienced progressive disease, resulting in a mPFS of 7.8 (4.7–10.8) months (**Figure 1**). The mPFS in patients with second line pyrotinib plus vinorelbine treatment was 12.0 (range, 3.8–20.2) months, and the mPFS for third-or-higher-line treatment was 6.4 (4.0–8.9) months. Only two patients received first-line treatment, which was not sufficient to calculate mPFS. The mPFS time was shorter for third-or-higher-line pyrotinib treatment than for second-line treatment; however, the difference was not significant (*p* = 0.225, **Figure 2**). Additionally, no significant difference was observed in mPFS between patients receiving pyrotinib plus oral vinorelbine or intravenous vinorelbine (7.8 vs. 6.4 months, *p* = 0.871; **Figure 3**).

**Figure 4:**
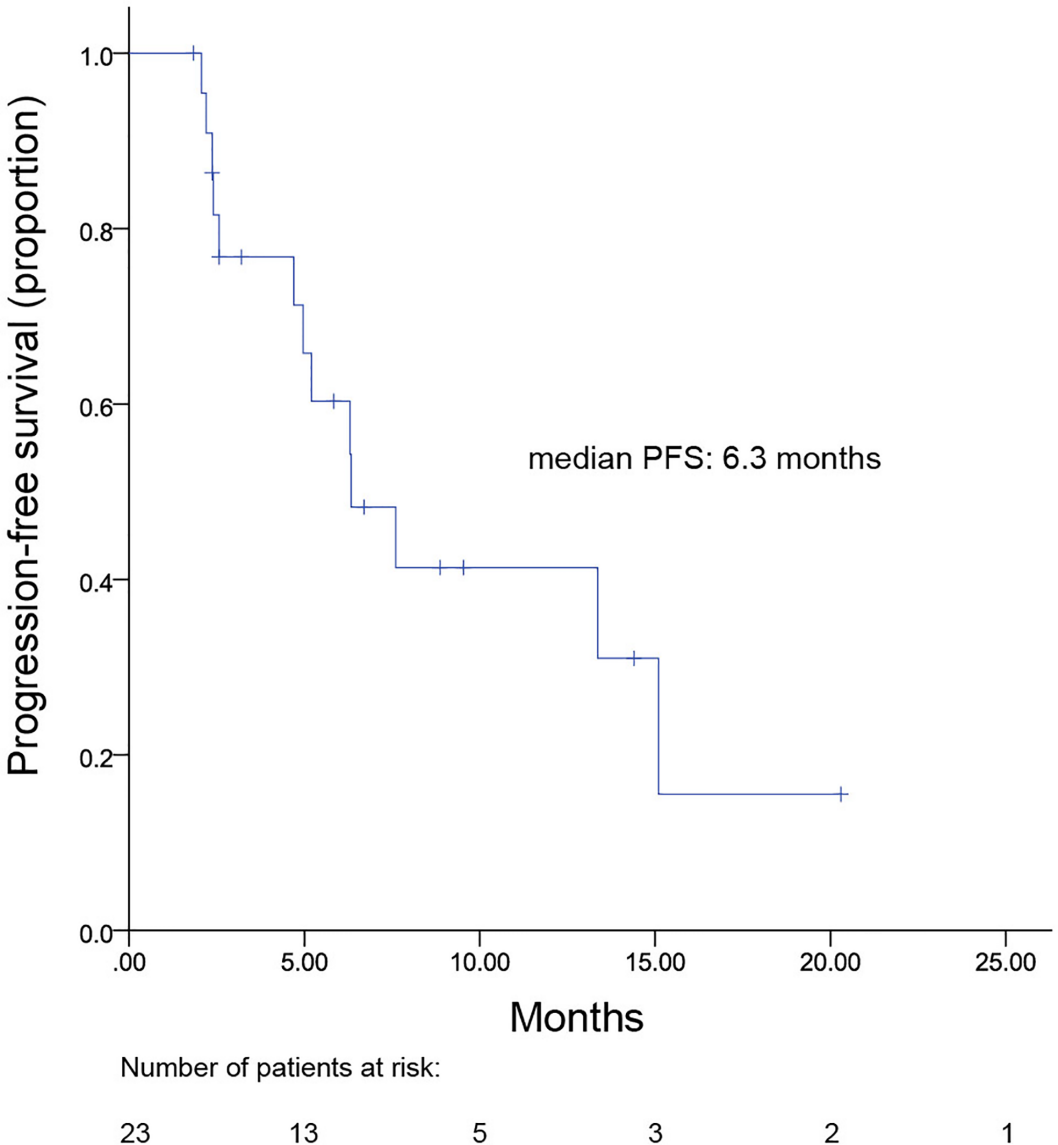
Kaplan–Meier plot for progression-free survival of patients with brain metastasis.

Overall mPFS (intracranial and extracranial lesions considered) for patients with brain metastases was 6.3 (range, 3.4–9.2) months (**Figure 4**). Meanwhile, no difference was observed in the PFS between patients with and without brain metastases (6.3 vs. 8.1 months, *p* = 0.825). OS data were not mature at the time of this report. A total of 96 patients were included in ORR analysis. No patient achieved CR and 33 had PR, resulting in an ORR of 34.3% (**Table 3**).

**Table 3:** Objective response rate in all patients.

Response	Number of patients (%) (N = 96)
Best response	
Complete response	0 (0)
Partial response	33 (34.3)
Stable disease	46 (48.0)
Progressive disease	17 (17.7)
Not evaluable	1 (1.0)
ORR	33 (34.3)

ORR, objective response rate.

**Figure 5:**
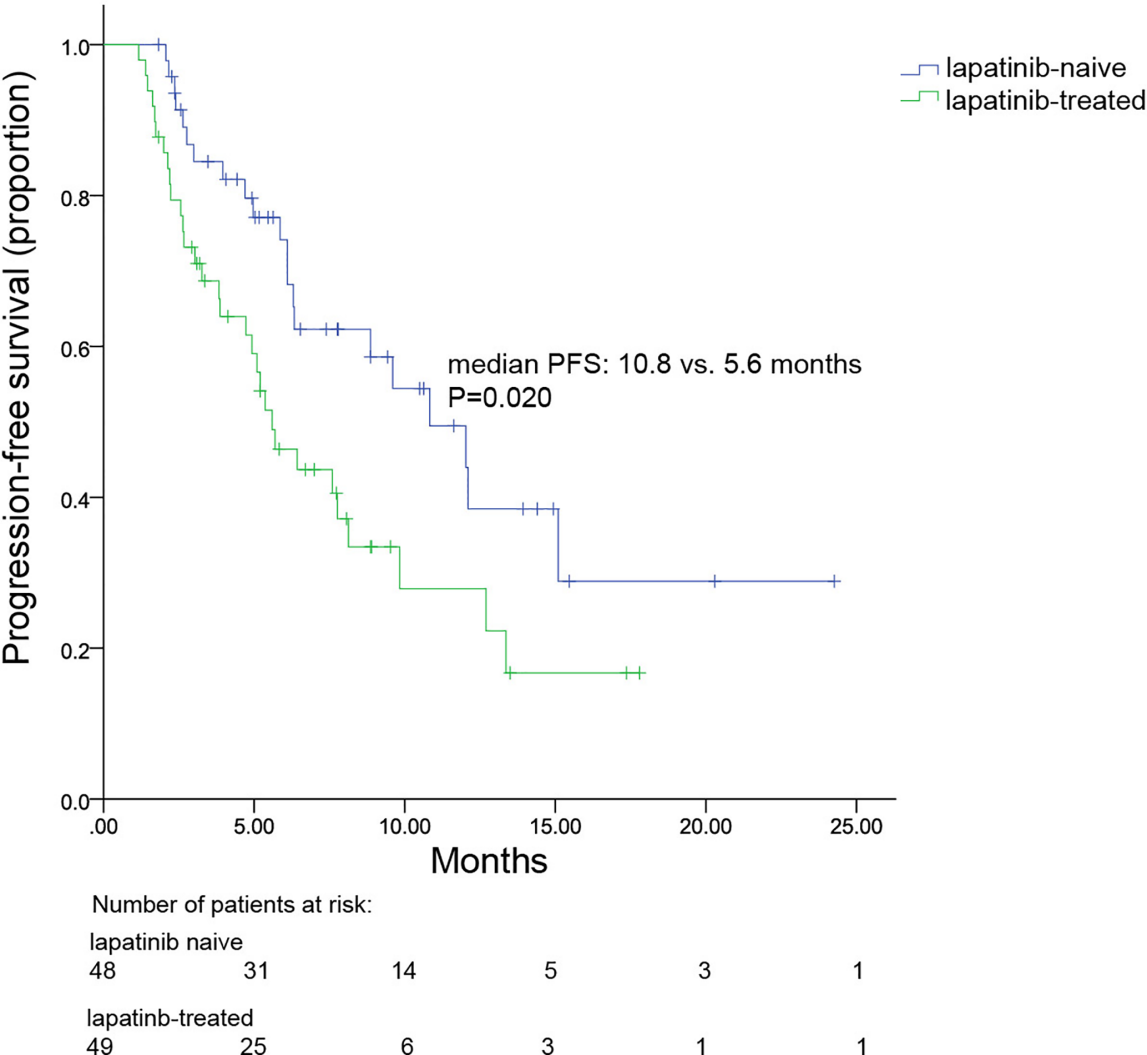
Kaplan–Meier plot for progression-free survival of patients with or without prior lapatinib exposure.

Univariate analysis indicated that age (< 50 years vs. ≥ 50 years), hormone receptor status (positive vs. negative), DFI (> 1 year vs. ≤ 1 year), number of metastatic sites (≤ 2 vs. > 2), types of metastases (visceral vs. non-visceral), lines of metastatic systematic therapy of pyrotinib plus vinorelbine (2 vs. ≥ 3), or trastuzumab resistance status (resistance vs. refractoriness) had no significant associations with mPFS in Log-rank analysis (**Table 4**). Only prior exposure to lapatinib (yes vs. no) was significantly correlated with mPFS in log-rank analysis (*p =* 0.020; **Table 4**). mPFS in patients with and without previous exposure to lapatinib were 5.6 months and 10.8 months, respectively (**Figure 5**). However, prior exposure was not an independent predictor of mPFS in Cox multivariate analysis.

**Table 4:** Log-rank and cox multivariate analysis of factors associated with progression-free survival.

Characteristic	HR (95% CI)	Log-rank analysis *p*-value	HR (95% CI)	Cox multivariate analysis *p*-value
Age group (< 60 years vs. ≥ 60 years)	0.856 (0.491–1.491)	0.583		
Hormone receptor status (positive vs. negative)	1.180 (0.679–2.052)	0.558		
DFI (> 1 year vs. ≤ 1 year)	0.938 (0.496–1.774)	0.843		
Number of metastatic sites (≤ 2 vs. > 2)	1.638 (0.887–3.026)	0.115		
Types of metastasis (visceral vs. non-visceral)	1.246 (0.683–2.272)	0.473		
Lines of advanced pyrotinib plus vinorelbine systematic therapy (2 vs. ≥ 3)	1.135 (0.815–1.579)	0.454		
Trastuzumab resistance status (resistance vs. refractoriness)	1.348 (0.754–2.410)	0.313		
Prior exposure to lapatinib (yes vs. no)	0.516 (0.296-0.901)	0.020	0.454 (0.200–1.031)	0.059

CI, confidence interval; DFI, disease-free interval; HR, hazard ratios.

### Safety

Because we collected information on AEs based on patients’ laboratory test results and medical records, and given the retrospective nature of the study, omission of AEs was unavoidable. Here, we report the grade 3 to 4 AEs, the most common of which were diarrhea (22.7%), neutropenia (7.2%), and leukopenia (4.1%; **Table 5**). No treatment-related death was reported. Overall, the safety of pyrotinib plus vinorelbine was controllable and tolerable.

**Table 5:** Grade 3 to 4 adverse events.

Grade 3 to 4 adverse events	Number of patients (%) (N = 97)
Diarrhea	22 (22.7)
Neutropenia	7 (7.2)
Leukopenia	4 (4.1)
Anemia	2 (2.0)
Thrombocytopenia	1 (1.0)
Nausea and vomiting	1 (1.0)
Fatigue	1 (1.0)
Weight loss	1 (1.0)

## Discussion

The advent of anti-HER2 therapy has greatly improved HER2-positive breast cancer prognosis. Specifically, pyrotinib is a novel anti-HER2 TKI that was recently approved in China. Our study showed promising effects of pyrotinib plus vinorelbine combination therapy with a median PFS of 7.8 months and an ORR of 34.3% in HER2+ MBC.

Evidence suggests that the strategy of trastuzumab plus pertuzumab combined with capecitabine for second-line treatment, maximizes mPFS to 11.1 months (21). Meanwhile, T-DM1 and lapatinib plus capecitabine have demonstrated a PFS of 9.6 and 8.4 months, respectively (4, 5, 22). In this study, the mPFS for patients with HER2+ MBC was observed to be 12.0 months and 6.4 months for second-line and third-or-higher-line pyrotinib plus vinorelbine treatments, respectively. Hence, pyrotinib plus vinorelbine could offer an alternative treatment as a second-or-higher-line treatment, to some extent.

However, our data were not as impressive as those for combined pyrotinib plus capecitabine reported in previous phase III trials with mPFSs of 11.1 and 12.5 months and ORRs of 68.6% and 67.2% (11, 12). Besides the obvious differences in chemotherapeutic drugs administered in combination with pyrotinib, the differences observed between our study and previous studies may have been caused by different population sample sizes and cohorts. For instance, the previous clinical trials included patients treated with no more than two lines of therapy, while some of the patients had not received any anti-HER2 therapy. However, in our cohort, 67% patients were treated with more than two lines of systematic therapy, and nearly all had received previous trastuzumab therapy. Therefore, our cohort represented a relative treatment refractory population, and the general population of HER2+ MBC patients in clinical practice were often heavily treated with multiple anti-HER2 agents. Hence, our study results provide data relevant to clinicians for the treatment of general metastatic HER2-positive BC patients in settings outside clinical trials. Additionally, the follow-up time for our study was relatively short (8.7 months) with more than 30% of patients still in treatment at the end of the period. Furthermore, the previous phase III trials excluded patients who were previously treated with lapatinib; meanwhile, 50.5% of patients in the present study were previously exposed to lapatinib. However, our study cohort included only a few patients who had been previously exposed to pertuzumab and/or T-DM1. Although pertuzumab and/or T-DM1 are commonly prescribed as front-line treatments for HER2-positive BC patients globally, in China, pertuzumab and T-DM1 was only newly approved, thereby limiting their usage in Chinese patients. Therefore, the role of pyrotinib in more heavily treated patients requires further global investigation.

Previous retrospective studies have also evaluated the efficacy and safety of pyrotinib-based regimens in real-world settings; however, the combination chemotherapy regimen used in these studies was mainly capecitabine (23, 24). A single-center retrospective study showed a mPFS of 6.3 months and an ORR of 29.5% in HER2+ MBC treated with pyrotinib-based treatment (23), whereas another multicenter analysis demonstrated an mPFS of 8.07 and ORR of 40.7% achieved by pyrotinib (24). However, few of the patients included in the two studies received combination pyrotinib and vinorelbine. Moreover, the mPFS for patients in second-line therapy was 12.0 months in our study, which is numerically higher than that reported for pyrotinib-based regimens in the previous multicenter retrospective study (8.1 months) (24), suggesting that pyrotinib plus vinorelbine is an effective treatment for HER2+ MBC, particularly as a second-line treatment.

The value of oral vinorelbine as a single agent for the treatment of MBC has been demonstrated in clinical trials indicating comparable efficacy and safety to intravenous vinorelbine. In our study, 74.2% and 25.8% of patients received oral vinorelbine and intravenous vinorelbine, respectively. Regardless of the drug formula, similar mPFSs were obtained when combined with pyrotinib. However, the oral formulation is easier to administer thus improving the quality of life in palliative settings and lowering the cost of medical care as it avoids hospitalization and reduces administration cost compared to the intravenous form (25, 26). Oral vinorelbine is thus a useful alternative to the intravenous form when combined with pyrotinib and deserves further clinical investigation.

Brain metastases frequently occur in HER2+ MBC compared to HER2 negative patients (1). For patients with brain metastasis, treatments are limited and prognosis remains poor, though anti-HER2 treatment was shown to improve survival in these patients (27–29). Meanwhile, the intracranial effect of trastuzumab remains controversial in brain metastasis patients as its large molecular structure hinders its ability to readily cross the blood-brain-barrier (BBB). Alternatively, anti-HER2 TKIs have become an important treatment strategy for these patients due to its small molecular size and high BBB penetrability. Indeed, a pooled analysis including 12 studies demonstrated that lapatinib plus capecitabine achieved s median pooled PFS of 4 months in HER2+MBC with brain metastasis (30). In the TBCRC022 trial, neratinib plus capecitabine also resulted in an mPFS of 5.5 and 3.1 months in lapatinib-naïve and lapatinib-treated HER2+ MBC patients with brain metastasis, respectively (31). Meanwhile, in our study, for the 23 patients with brain metastases, the mPFS was 6.3 months. Similarly, within the PHENIX study, a subgroup of patients with brain metastases had an mPFS of 6.9 months following combinatorial treatment with pyrotinib and capecitabine (11). However, considering the small number of brain metastases patients, larger scale clinical trials are warranted to verify the effectiveness of pyrotinib plus vinorelbine in patients with brain metastases.

Our results also revealed that the efficacy of pyrotinib plus vinorelbine therapy was significantly higher in lapatinib-naïve patients than in lapatinib-treated patients. In lapatinib-naïve group, pyrotinib plus vinorelbine therapy achieved an mPFS of 10.8 months, while a shorter mPFS of 5.6 months was observed in the lapatinib-treated group. In fact, the mPFS for the lapatinib-naïve group was numerically comparable to that of a neratinib plus capecitabine group (8.8 months), and better than that of a lapatinib plus capecitabine group (6.6 months) reported previously in the NALA study (22). Additionally, the mPFS in the lapatinib-treated group was numerically higher than that reported in the TBCRC022 trial (3.1 months) (31). Therefore, this is the first study, to our knowledge, to demonstrate the effectiveness of pyrotinib plus vinorelbine in patients following the failure of lapatinib-based treatment.

Pyrotinib plus vinorelbine therapy was generally well-tolerated with diarrhea found to be the most common grade 3 to 4 AE in the present study, which was consistent with reports of previous clinical trials (11, 12). However, all AEs were effectively controlled with treatment and did not lead to discontinuation of pyrotinib or vinorelbine treatment during the study. Notably, incidences of grade 3 or 4 neutropenia and leukopenia were present in 7.2% and 4.1% of patients in our study, respectively, which represented higher incidence than that reported in previous phase III clinical trials (11, 12), which was likely the result of the patients in our study being treated in combination with vinorelbine. In addition, no grade 3 to 4 hand-foot syndrome was reported, likely because no patients received capecitabine as a combined therapy. However, due to the retrospective nature of the study, missed AEs was unavoidable.

The current study has certain limitations. First, the retrospective and observational nature of the study may have resulted in missing data or possible recall and information bias. Second, the length of follow-up was relatively short and insufficient to allow for OS conclusions to be made. Nevertheless, our study also had certain associated strengths. First, it provides evidence to support the efficacy of combinatorial treatment with pyrotinib plus vinorelbine in real-world setting. Second, to our knowledge, this represents the first, and largest, observational case series available thus far. Finally, our results report the treatment pattern and safety data for pyrotinib plus vinorelbine in clinical practice, providing a theoretical basis for clinicians.

## Conclusions

The combination of pyrotinib plus vinorelbine therapy demonstrated promising effects in HER2+ MBC with tolerable toxicity, particularly in patients administered the combination as a second-line treatment, and in those without prior lapatinib treatment. Pyrotinib plus vinorelbine also demonstrated promising anti-tumoral activity in patients with brain metastases. Additionally, oral vinorelbine offers a useful alternative to the intravenous form when combined with pyrotinib. However, additional clinical trials are required to further exploit the potential of pyrotinib plus vinorelbine.

## Data Availability Statement

The original contributions presented in the study are included in the article/supplementary material. Further inquiries can be directed to the corresponding authors.

## Ethics Statement

The studies involving human participants were reviewed and approved by Fudan University Shanghai Cancer Center. The patients/participants provided their written informed consent to participate in this study. Written informed consent was obtained from the individual(s) for the publication of any potentially identifiable images or data included in this article.

## Author Contributions

BW and RG conceived and designed the study. YL, HL, TL, WL, HW, and BS collected the data. YL and YQ performed the statistical analyses. YL wrote the manuscript. BW and RG reviewed and revised the manuscript. All authors contributed to the article and approved the submitted version.

## Funding

This study was funded by the National Natural Science Foundation of China (NSFC) [Grant No. 81874114] and Shanghai "Rising Stars of Medical Talent" Youth Development Program [Grant No. AB83190002012023].

## Conflict of Interest

The authors declare that the research was conducted in the absence of any commercial or financial relationships that could be construed as a potential conflict of interest.
